# Medical and Physical Intelligence

**Published:** 1802-12-01

**Authors:** 


### MEDICAL AND PHYSICAL

### INTELLIGENCE.

### [FOREIGN AND DOMESTIC.]

---

The Managers of the Vaccine Pock Inftitution for inoculating the poor, at No. 5, Golden Square, have circulated an Addrefs on the fubject of the new Inoculation; from which, on account of its perfpicuity and peculiar force, we have felected fome paffages.

" Those who are acquainted with only a part of the hiftory of the Small-Pox, fcarcely take into their contemplation more than the advantages of the *inoculated* over the *natural Small-Pox,* in the points of prefervation of the lives of individuals, and the fubftitution of a difeafe generally flight, for a difeafe generally fevere: and fuch perfons imagine, that the practice of Inoculation neither requires, nor is, perhaps, capable of farther improvement. But thofe who are more extenfively acquainted with the hiftory of the Small-Pox, know, that it is productive of a great deal of mifchief, notwithftanding the advantages of Inoculation—For,

 " Under

"Under the beſt treatment, a certain proportion of perſons die in the inoculated Small-Pox; and although the proportion of deaths to the recoveries may not exceed Five out of a Thouſand Patients, the diſtreſs occaſioned by theſe fatal caſes is more ſeverely felt than when ſuch caſes occur in the caſual diſeaſe: therefore the ſubſtitution of a milder diſeaſe will contribute to leſſen the diſtreſs which would thereby be occaſioned.

"It ſeems fair to calculate, that, in the inoculated Small-Pox, one in twenty-five patients undergoes a ſevere illneſs.

"The numerous ſources of the Small-Pox infection now preclude every proſpect of extinguiſhing this malady; and unleſs Inoculation were univerſally practiſed, it is moſt probable that the proportional mortality by the natural Small-Pox is rather increaſed than diminiſhed, in conſequence of the more extenſive diſſemination of the infection by Inoculation.

"In a certain proportion of inoculated caſes of Small-Pox, deformities of the ſkin are produced by the eruptions, which no practitioner can be anſwerable for preventing in any inſtance. Diſeaſes alſo are ſometimes excited by inoculation, to which a diſpoſition pre-exiſted in the conſtitution.

"In particular families, and in particular ſtates of the conſtitution, as in pregnancy, &c. the Small-Pox are an exceedingly dangerous diſeaſe, even by inoculation. Now it is manifeſt, from the accounts which have been collected of the diſorder called by the name of the Cow-Pock, and particularly from the experience by inoculation of it ſince January, 1799, that the hurtful effects of the Small-Pox above ſtated may be prevented, by ſubſtituting for it the inoculation of the Cow-Pock — Becauſe,

"1. Of above one hundred thouſand perſons in Europe who have had the inoculated Cow-Pock, it is very doubtful whether any one has died of the inoculation. There is, however, good ground for believing, that the fatal caſes reported by inoculation, in ſome inſtances, were from the interference or ſupervention of other diſeaſes; and in other inſtances, the reports, on enquiry, were found to be groundleſs, no one having died.

"2. Not a ſingle well-atteſted inſtance has been produced, among more than 50,000 of the above perſons known to have been conſtitutionally affected with the inoculated Vaccine Pock, and who were ſubſequently inoculated for the Small-Pox, of this diſeaſe being taken, and in thoſe few inſtances which have been ſuppoſed caſes to the contrary, there was every reaſon to believe the inoculated Diſeaſe was local, or not the real diſorder; and traditionally, this fact has been eſtabliſhed time immemorial, with regard to the caſual Cow-Pock.

"3. It may ſafely be affirmed, that the inoculated Cow-Pock is generally a much ſlighter diſeaſe than the inoculated Small-Pox; and that the proportion of ſevere caſes in the latter, is to the former as at leaſt a hundred to one

"4. It does not appear that the genuine Vaccine Pock can be propagated like the Small-Pox, by effluvia from perſons labouring under it.

it. Hence, if the Vaccine Inoculation ſhould be univerſally inſtituted in place of the Small-Pox, it is reaſonable to conclude, that this moſt loathſome and fatal malady will be extinguiſhed; and like the Sweating Sickneſs, Plague, certain kinds of Leproſy, &c. be known in this country only by name.

" 5. It does not appear that the Vaccine effluvia, like the Small-Pox, can be conveyed ſo as to produce the diſeaſe indirectly from diſeaſed perſons, by adhering to clothes, furniture, bedding, letters, &c. Hence no danger of its propagation in theſe channels is to be apprehended from the univerſal practice of implantation of the Cow-Pock. Further, it is highly important to conſider other very great advantages of the Vaccine Inoculation over that of the Small-Pox, which are, beſides thoſe already mentioned, that it may be practiſed in the ſtate of pregnancy, in infancy, and in all ages, generally with little or no inconvenience, being almoſt always a perfectly mild diſeaſe. It may be practiſed on a part of a family, without any danger of infecting the reſt; and of courſe it may be introduced into places and towns to any extent, not only without endangering thoſe undergoing the diſeaſe, but, from its not being infectious by effluvia, without any danger of communicating it to others who have not had the Small-Pox. And, even at this day, in ſome places, the Small-Pox being looked upon as a plague, the Inoculation is prohibited under the ſtricteſt regulations; but if the advantages of the new Inoculation ſhould be underſtood by people ſo circumſtanced, it is hardly to be doubted that the Vaccine Inoculation will, on the firſt breaking out of the Small-Pox, be adopted.

" 6. No danger is to be apprehended from the interference of the Small-Pox; for it has been abundantly proved, that if a perſon is infected with Small-Pox effluvia previous to the Cow-Pock Inoculation, and the Small-Pox takes place at the ſame time with the Cow-Pock, that the former diſeaſe is commonly mitigated by the latter. And there is now good evidence to ſhew, that variolous like eruptions never appear, except on thoſe ſubjects who previouſly to the inoculation for the Cow-Pock had been expoſed to variolous effluvia.

" 7. Experience ſhews, that there is no reaſon to apprehend the ſmalleſt chance of deformities of the ſkin from the Cow-Pock Inoculation.

" 8. The extenſive practice of the new Inoculation in the preſent and the two former years, and the accounts of the diſeaſe in the caſual way, do not ſhew that any other diſeaſe will be excited ſubſequently, which is peculiarly imputable to the new practice.

" It may be uſeful to add, that the preſent Inſtitution is perhaps the beſt imaginable for procuring evidence to inform thoſe who are unacquainted with the new practice; for determining all doubtful points relating to it, and for diſcovering errors; as every caſe will be regiſtered; every improvement be made under the direction of the Medical Eſtabliſhment belonging to the Inſtitution; and the reſults of the practice will be reported to the Governors.

" From the above comparative ſtatement, it is manifeſt that it is

 highly highly to the intereſt of the public to adopt univerſally the Inoculation of the Vaccine Pock in place of the Small-Pox."

### Bloomsbury Dispensary.

The Vaccine Inoculation is practiſed gratuitouſly, and without any recommendation, at the *Bloomsbury Dispensary*, Great Ruſſell Street; where medical Gentlemen may obtain the virus, properly authenticated, every Saturday at ONE O'CLOCK PRECISELY, by applying to any of the medical Officers of the Inſtitution, or to Dr. JENNER, who ſuperintends the inoculating department.

A correſpondent has favoured us with the following NOSOLOGICAL DESCRIPTION OF THE COW-POCK:—THE COW-POCK, he obſerves, is a circumſcribed, elevated, ſolitary Veſicle, nearly circular at its baſis; having a regular, ſmooth circumference, but is flattened in its apex; ſurrounded, about the tenth or twelfth day, by an eryſipelatous efflorefcence; afterwards desiccating into a browniſh, hard, gliſtening incruſtation, which ſoon falls off, and leaves a white cicatrix through life.

### *Preparation of the Lichen Islandicus.*

Mr. RICHARD REECE, of Henrietta-ſtreet, Covent-garden, writes to us, that, having obſerved in the laſt Number our notice of Dr. Regnault's "Eſſay on the Lichen Iſlandicus in pulmonary Conſumption," he thought the information where the genuine herb may be obtained, would not be unacceptable to our readers, particularly, as he believes the liverwort generally ſold by herbaliſts to be the produce of this country. From the high character given of the dietetic and medicinal properties of this ſpecies of Lichen in Phthiſis Pulmonalis, by Prof. Murray, and ſeveral eminent practitioners on the Continent, and Dr. Crichton in this country; Mr. R. was induced, ſome time ſince, to procure a quantity from a Correſpondent on the Continent, and from the few experiments that he has made with it, he is convinced that it poſſeſſes very conſiderable anti-phthiſical powers. He is now trying it in the manner recommended by Dr. Regnault, in the form of a concentrated ſyrup, of which he has prepared a quantity for the uſe of thoſe phyſicians who chuſe to preſcribe it.

From the favourable reports of the deobſtruent properties of the Rubia Tinctorum in Chloroſis, by Prof. Home of Edinburgh, and the great ſucceſs that attended its exhibition in thoſe caſes under the direction of Dr. Symonds and Dr. Blount, during Mr. R's reſidence as domeſtic Surgeon and Apothecary at the General Infirmary at Hereford, he was induced, ſome time ſince, to make an extract from a cold infuſion of the root, in the ſame manner as recommended by the French chemiſt for the *Sel essentiel de Cinchone*, communicated in a former Number of our Miſcellany.

This

This extract contains, in a small bulk, the active properties of the root, and being free from its indissoluble matter, may be administered in sufficient quantity after the full dose of the powder recommended by Dr. Home has proved obnoxious to the stomach of a delicate female. Besides, continues Mr. R, the powder being prepared for the use of dyers, is seldom to be obtained sufficiently fine for medicinal purposes. The extract may be exhibited in a pilular form, or dissolved in mint water in the proportion of two drachms to half a pint, and two or three table spoonfuls taken three times a day. The latter is rather a pleasant medicine than otherwise. Dr. Osborne, in his Lectures on Midwifery, speaks very highly of the ecphratic properties of the rubia tinctorum, and relates a case of chlorosis, in which it proved eminently serviceable in correcting the scrophulous diathesis with which the patient was afflicted.

Mr. Reece states, that he has received the testimonies of several eminent practitioners of the great advantages of the essential salt of bark over the powder in typhus and intermittent fevers, in being retained in the stomach after the latter has been rejected, and that it has in no instance excited vomiting or diarrhœa. A Surgeon of considerable eminence in his neighbourhood, afflicted with ague, has received every advantage from it after the powder, in every form he could conceive, was rejected.

---

*A new Method of preparing Phosphoric Ether, by* Cit. Boudet.

The combination of phosphoric acid with alcohol seems to have been hitherto little regarded by chemists, as the authors who have written on ethers in general, make no mention of the phosphoric ether. In order to supply this defect, Cit. *Boudet* undertook a series of experiments, the results of which are here communicated.

The success of the experiments depending, in a great measure, on the purity of the phosphoric acid, he endeavoured to obtain it in as pure a state as he possibly could, for which purpose he proceeded in the following manner.

A glass balloon, of a considerable capacity, was connected with the neck of a large tubulated retort; the balloon communicated with Woulf's apparatus, the last receiver of which was in contact with the external air, by means of a long tube, which passed through the window of the laboratory. Having properly secured the junctures of the vessels, he poured into the retort six parts of nitric acid, purified in the common way, the weight of which, to distilled water, was nearly as 8 : 80. When the liquor was, by a proper heat, brought to the boiling point, one part of phosphorus, cut in pieces, was introduced into the retort, through its tubulus: every time a piece was thrown in, a great quantity of a reddish, more or less, dark gas disengaged itself, and it was not thought proper to put in another piece, until the gas had ceased to be disengaged. In this way the author proceeded, till all the phosphorus was brought in. It is remarkable that the retort was always always placed in the fand bath, in fuch a way as to form, with its neck, an obtufe angle, by which means the too hafty concentration of the nitric acid was prevented; a circumftance which is very much to be dreaded in this operation, becaufe the nitric acid being too much concentrated, fuffers the phofphorus to fwim on its furface, where it burns, but only on the furface, and may thus occafion the cracking of the veffels. After the deflagration of the phofphorus is finifhed, the liquor contained in the retort is to be poured into a matrafs or phial, which being placed in the fand bath, is thus far heated, till all the nitric fumes are diffipated. The liquor being cooled, a more or lefs thick fubftance will be obtained, which may be confidered as a good phofphoric acid, poffeffing all the requifite characteriftic qualities. With this acid the author undertook the following experiments, for the purpofe of obtaining phofphoric ether.

A tubulated retort was placed in the fand bath, combined with a balloon, which was likewife provided with a tubulus, by which it was connected with one adopter of Woulf's apparatus, which had been previoufly filled to two-thirds with lime water. Into this adopter a glafs tube was applied, which made a combination between this and the pneumatic apparatus. The veffels being put in proper order, and their junctures well luted, the author mixed, in a glafs mortar, equal parts of the above phofphoric acid, and of very pure alcohol of 38° of Beaume's areometer, at 14° of Reaumur; the moment the mixture was made, a confiderable quantity of calorique was difengaged, capable of making the thermometer rife above 14.° The mixture was then brought into the retort, and being gently heated, it began to boil at a temparature of 60°. In this ftate it was fuffered to remain fome time, to give an opportunity of examining every thing that might happen during the operation. At firft an uncoloured liquor paffed over in the receiver, which fmelled like fpirit of wine, and which was feparated by means of a fyringe. The next liquor which paffed over was alfo without colour, but of a different fmell. During this time, the mafs remaining in the retort acquired a brownifh colour, which became darker during the continuance of the diftillation. When this liquor had obtained a fort of confiftency, the operation was difcontinued, in order to feparate the fecond product; but after having replaced the veffels in due order, the fire was carefully increafed, till the remaining mafs appeared to puff up, and to pafs over.

In this manner, he obtained—1. An acid, faintly-coloured liquor of a difagreeable fmell, which refembled the refiduum remaining after the diftillation of bituminous fubftances. 2. An oil, which had in the beginning a faint yellowifh colour, but became darker by degrees; it fwam on an acid liquor. Thefe two liquors paffed over with an elaftic fluid, which was carefully collected. Lime water, through which it was fuffered to pafs, did not become turbid. On touching it with a light, it burned without detonation with a blue flame, which however appeared white in the glafs bell, on the walls of of which it depofed a mafs perfectly fimilar to foot, whence the author judged this fluid to be hydrogen, overcharged with carbon. The fire was now increafed to fuch a point, that the retort began to melt, and the product obtained by this degree of fire was a much thicker and browner oil, part of which fell to the bottom of the liquor contained in the receiver. It was likewife obferved, that a footy matter had been precipitated in the neck of the retort, in form of globules, which, on being fqueezed, had a fomewhat metallic appearance. In order to learn the contents of what had remained in the retort, it was broke into pieces, upon which a blackifh matter, intermixed with white opacous particles, was difcovered. The weight of this mafs was confiderably lefs than the phofphoric acid which had been employed; when expofed to the atmofpherical air, it attracted moifture; and being thus liquefied, it became a very acid liquor, which being faturated with carbonat of foda, yielded cryftals of phofphat of foda, which proved its being nothing but phofphoric acid.

The products remained now to be explored, on which account the firft liquor that had paffed over was examined: from its colour, fmell, inflammability, and from the eafy combination with water, it proved to be fpirits of wine. The fecond product, however, had the fmell of ether, a little mixed with a fmell analogous to that of garlick: blue vegetable colours were faintly reddened by it. Conceiving that it was not fufficiently pure, the author endeavoured to purify it by diftilling it over carbonat of magnefia. During this procefs, carbonic acid was difengaged, and the liquor paffed over before it came to the boiling point. When about one-third of the liquor had been diftilled over, the heat was diminifhed, and the apparatus being cooled and the veffels feparated from each other, the liquor was examined. It was without colour, volatile, and its fmell refembled that of ether vitrioli; its deflagration was violent without leaving any foot; it was lighter than water, on which it fwam, but on being ftirred together, it could be mixed with it; when burned on water it left no refiduum. It diffolved volatile oils, but appeared to have no effect on the fixed oils: it had a confiderable effect on phofphorus, which immediately imparted to it the peculiar phofphoric fmell. Its weight, compared with alcohol, was as 94 : 100; with diftilled water, as 88 : 41; with vitriolic ether, as 426 : 482; or as 213 : 241. By thefe experiments, it appears to be proved, that this liquor is a true ether, the properties of which deferve to be farther examined by repeated experiments, particularly as they feem to deviate in fome refpects from thofe afcribed to ethers in general.

*Note.* Cit. *Boudet* feems not to have been acquainted with the experiments which the French as well as German chemifts have made on the combination of the phofphoric acid with alcohol. *Guyton de Morveau* fays, in his Elements of Chemiftry, that fpirits of wine diftilled over phofphoric acid carry over a part of the latter. *Lavoifier*, on diftilling alcohol over ftrong phofphoric acid, obtained ed a liquor of an agreeable ether-like fmell: *Cornette* obtained, by the diftillation of alcohol over ftrong phofphoric acid, a dulcified fpirit, refembling a true ether. Mr. *Weftrumb* obtained it in a more perfect ftate by diftilling alcohol over phofphoric acid and manganefe: it had a fmell analogous to that of the pear of the quince tree, (pyrus cydonia), and on being burned on the furface of water it left behind the fmell of garlick.

---

Mr. CUTHBERTSON gives the following account of an experiment, by which the two kinds of electricity are diftinguifhed, or the direction of the fluid is afcertained: Infulate two wires, furnifhed at each end with a ball, three-fourths of an inch in diameter; connect one with the pofitive, and the other with the negative conductor of a machine; the balls fhould be four inches afunder, and between them, at equal diftances from each place, a lighted candle, with the centre of its flame nearly on a level with the centres of the balls. If the machine be put into motion, the flame will waver very much, and feem to incline rather more to the negative ball than to the pofitive one; after about fifty revolutions, the negative ball will grow warm, and the pofitive ball remain cold; if the revolution be continued to about 202, the negative ball will be too hot for the hand to touch, while the other remains as cold as at the beginning.

---

Citizen HAUY having compared the methods of correcting and calculating of feveral celebrated blind men, has digefted into a body of doctrine the beft productions of experience in this art.— His method of *writing* confifts in ufing an iron pen, the point of which is not fplit. By writing without ink, and preffing on a ftrong paper, the blind man produces a character in relief, which he can immediately read by paffing his fingers over the projecting characters on the oppofite fide of the paper, in the contrary direction. The relief is fufficient, provided a foft furface be placed under the paper, fuch as leather, blotting paper, &c.

---

Citizen PICTET gives an account of experiments, to prove that light and heat are *not* the fame. Oppofite to each other he places two concave metallic mirrors; in the focus of one he places a lighted candle, and in the focus of the other a very fenfible air thermometer: he then places between the foci, a piece of very thin and tranfparent glafs; the thermometer, indicating the tranfmiffion of heat, ftopped that inftant. The two mirrors were placed at the diftance of about twenty-five yards one from the other, in order to determine whether the time of the propagation of the radiant heat, from one focus to the other, could be appreciated. A heated, but not luminous ball, was fufpended at one of the foci, before which a fcreen was placed. At the inftant that this obftacle was removed, the fluid in the thermometer, which was before perfectly at reft, began to move, and no fenfible interval could be perceived between the fuppreffion and the effects of the tranfmiffion of heat.

---

From

From ſome late experiments made by Mr. FRANCILLON, it appears, that a mixture, conſiſting of ſix parts of gold, and one of platina, gives a metal of a beautiful colour, great malleability, and capable of receiving an exceedingly fine poliſh, more unalterable than gold, when expoſed to the action of ſulphuriſed hydrogen, and other agents.

---

A new metallic ſubſtance has lately been diſcovered in Sweden. The ore has a blackiſh colour, with the metallic aſpect of cryſtals of oxidated tin; its colour is equally dark; its gravity is conſiderable; it ſtrongly ſcratches glaſs. M. EKEBURG has extracted from this mineral a new metallic ſubſtance, to which he gives the name of Tantaliti.

---

From ſome experiments in electricity, Citizen TREMERY concludes, that the atmoſpheric air, in its ordinary ſtate, reſiſts the paſſage of the negative, more than the poſitive, fluid, and that the inſulating property of non-conductors cannot be the ſame for both electricities.

---

The vaccine inoculation continues to make rapid progreſs in Spain and Italy. In Catalonia 7000 perſons were inoculated in the courſe of nine months; and, by its means, the fatal ravages of the ſmall-pox have been ſtopped in the department of Milla, where, during three months only, 12000 perſons have ſubmitted to the vaccine operation.

---

Cit. SEQUIN, an Aſſociate Member of the National Inſtitute, lately read two Memoirs relative to *Cinnaber, (cinoper* or *vermillion)* in which that chemiſt endeavours to prove, that *ethops* and *cinibe*r are only a compoſition of ſulphur and of mercury, without oxygen and without hydrogen; that theſe two ſubſtances only differ from one another in the proportion of their principles or conſtituent parts, and, above all, in the degree of union of their molecules or particles; that this proportion, and this degree of union, are invariable in cinnaber; and on the contrary, very variable in ethiops; and, laſtly, that cinnaber is a compound of thirteen parts and one-third of ſulphur, and of eighty-ſix parts and two-thirds of mercury.

---

Mr. EZEKIEL WALKER has diſcovered a cheap method of producing light, which he thinks poſſeſſes advantages much ſuperior to the common modes of illumination. This light generates no ſmoke, nor does it require the aid of ſnuffers.

---

### *An advantageous Method of preparing the Red Oxyd of Mercury.* *By* I. W. C. FISCHER.

Cit. Van Mons having remarked, that in preparing the red oxyd of mercury from the nitrat of mercury, a conſiderable quantity of the acid employed for the oxydation of the mercury is diſengaged on heating heating the nitrat of mercury, propofes to take a greater portion of mercury than the quantity of acid employed in this procefs is able to diffolve, becaufe the fuperabundant mercury will be likewife oxydated by the acid that is difengaged on heating the faid nitrat. The nitric acid however being of a different degree of concentration in the laboratories, I propofed, fays M. Fifcher, for the purpofe of finding the juft proportion of mercury, to rub the dry and hot prepared nitrat oxyd of mercury with one third or one-half of metallic quickfilver, and to expofe it to the common treatment in fire, by which means a certain proportion may be found out. Mr. Schmidt, apothecary, at Sonderburg, made feveral experiments according to the prefcription of Van Mons, but without the intended fuccefs, as the quickfilver, which had been employed in a fuperabundant quantity, was obtained again in a metallic form. The refults of my experiments however have anfwered my moft fanguine expectations.—Four hundred parts of metallic quickfilver were diffolved in nitric acid at the heat of boiling water; in order to obtain a thoroughly neutralized folution, the acid was added by drops till all the quickfilver was diffolved. The folution being evaporated to drynefs, the dry falt thus obtained is rubbed with 350 parts of metallic quickfilver. The powder received a dark grey colour, but on being mixed with a little water to the confiftency of a bolus, for the purpofe of producing a more perfect union of the metallic quickfilver, the colour became greyifh white, and it only required a few minutes rubbing to make the metallic quickfilver entirely difappear. The moift mafs having been gently dried, was put into a retort and expofed to a gradual heat. Three minutes after, oxygen gas was difengaged, and the whole mafs began to change its greyifh colour into dark red. The procefs having been finifhed, the mafs after being cooled, fhowed in all its parts a light red colour, and was reduced to the fineft powder, which could entirely be taken out of the retort without breaking the veffel into pieces. There were no traces of metallic quickfilver in the receiver, nothing but a fmall quantity of acid fluid having paffed over, which induced me to repeat the experiment with a ftill greater quantity of quickfilver. To 400 parts of quickfilver, diffolved in the above manner by nitric acid, and afterwards evaporated, I added 400 parts of metallic quickfilver, and treated the mixture as before; the refults, however, were about the fame as in the former experiment.

From thefe experiments, it appears, that by the method which is here propofed, not only half the quantity of nitric acid is faved, but the retort which has been ufed may be employed feveral times to the fame purpofe. Another advantage of this method confifts in obtaining the red precipitate in form of a fine powder, which faves a great deal of trouble. The time and fire, required in this method, are by far much lefs than in all others, as in lefs than thirty minutes feveral pounds of the above white mercurial bolus may be changed into red oxyd of mercury. The procefs with fome ounces was always finifhed within four or five minutes. On account of all thefe advantages, this method deferves to be particularly recommended.

It

It is not unknown to the readers of this Journal, that Sig. Gerbi, an Italian gentleman, was the firft who difcovered the fingular efficacy of a certain infect, when applied as a remedy for the tooth ach; a fact, which was afterwards confirmed by Dr. Corradori, by whom feveral other infects were found to prove equally efficacious in the fame complaint; for inftance, the *coccinella feptem-punctata, &c.* This infect alfo was highly recommended by a certain German dentift, as an excellent anodyne in feveral kinds of tooth ach. Dr. Sauter likewife extols the efficacy of this fpecies of coccinella in mitigating the pains of affected teeth; and he obferves, that on applying it to the tooth, a particular fenfation of cold is perceived, which always precedes the ceffation of the pain. He alfo remarks, that the infect emits a ftrong odour of opium. In order to preferve the infects in winter, it is advifable to put them into a box, perforated with fmall holes and filled with earth, which muft, from time to time, be moiftened, and to give them a little grafs or hay to feed on. The fmell of opium which they emit, feems to be ftronger in winter than in fummer. Dr. Sauter made feveral experiments for the purpofe of fixing the efficacious particles of this infect, as, in form of a conferve, &c. which however did not fucceed. At laft he bethought himfelf that the efficacious particles might be extracted by fpirit of wine, and prepared accordingly the following tincture. Sixty or eighty fpecimens of the coccinella feptem-punctata were fqueezed in a ftone mortar, and being triturated with one ounce of fpirit. vini rectific. the mixture is expofed during eight days to funlight, being fhaken from time to time. Afterwards it is expreffed, filtrated, and muft be kept in clofe veffels. This tincture has a yellow reddifh colour in winter, and changes the colour to red in fummer; a white fediment falls generally down to the bottom. In this form, it was eafy to bring the efficacious particles into the body; and Dr. S. being curious to know what effect it might have when ufed internally in painful affections, he determined to take the firft opportunity of trying it. This was foon offered in the cafe of a lady, who having frequently had a rheumatic tooth ach, contracted, foon after her delivery, a violent pain in the jaws, which particularly attacked her in the night, and againft which opiates, extract. aconiti had been employed, without producing much effect. At laft he had recourfe to the tincture coccinella, of which fhe was ordered to take 20 drops in the morning and in the evening; but being one night attacked by a violent fit of the pain, fhe fwallowed above half a table fpoonful of the tincture, after which the pain began gradually to abate, and ceafed in a few minutes. Having continued the tincture in a larger dofe, fhe was entirely cured of that painful affection. This remedy proved likewife ferviceable in a cafe of hæmicrania, and it even produced relief in a cafe of the Fothergillian face ach, (tic douloureux.)

---

Mr. Thomas Salmon, of Canterbury, has given a defcription of a fimple method for clearing apartments from noxious air. He carries carries an air tight metallic tube from the upper part of the place in which the noxious air is generated, as common-fewers, cefs-pools, privies, &c. with an afcent towards the kitchen, or other chimney, whofe fire is moft frequently kept, and joined to the lower part of the back of the grate; a pipe is alfo fixed at the upper part of the grate, which is made to conduct the neareft way out of the houfe. By this method, Mr. Salmon fays, holds of fhips may be ventilated without labour or expence, by paffing the metal pipe through the cabin or other fire; and that deftruction of grain prevented that was experienced during the late fcarcity.

---

Dr. FORBES, of the Univerfity of Edinburgh, continues to devote his attention to the improvement of Medical Gentlemen in the Latin language, previous to Graduation. The method of teaching which he has hitherto purfued, and to which his invariable fuccefs determines him to adhere, he refpectfully fubmits to the confideration of fuch gentlemen as may, during this feafon, be inclined to employ him. He converfes with his Pupils in the Latin language, on the anatomy and phyfiology of all the vifcera contained in the different cavities of the human body; on the difeafes to which thefe parts are fubject; on the pathology of thefe difeafes; on their diagnoftic and prognoftic fymptoms, and on the moft rational indications of cure, with conftant reference to the moft approved remedies. He takes a view of the origin, the diftribution, and the economy of the nervous, the fanguiferous, and the abforbent fyftems. He confiders fome of the moft interefting proceffes of the animal economy, fuch as the vital functions of circulation and refpiration, the natural ones of digeftion, fecretion, &c. in the light in which the prefent improved ftate of Chemiftry enables us to view them. He unfolds the chemical analyfis of the fluids, and ftates the moft material changes which they undergo by difeafes. He pays due attention to the Materia Medica, and to Chemical Pharmacy. Dr. F. reads any Medical Latin Author, with whom Gentlemen may wifh to become better acquainted; and makes fuch obfervations on the language, or fcope of the Author, as the portion perufed may fuggeft; he alfo affifts his Pupils, either by his advice or corrections, in bringing forward their Inaugural Differtations.

